# Aberrant methylation of LINE-1, SLIT2, MAL and IGFBP7 in non-small cell lung cancer

**DOI:** 10.3892/or.2013.2266

**Published:** 2013-01-31

**Authors:** MAKOTO SUZUKI, KENJI SHIRAISHI, AYAMI EGUCHI, KOEI IKEDA, TAKESHI MORI, KENTARO YOSHIMOTO, YASUOMI OHBA, TATSUYA YAMADA, TAKAAKI ITO, YOSHIFUMI BABA, HIDEO BABA

**Affiliations:** 1 Department of Thoracic Surgery, Kumamoto University, Kumamoto 860-8556, Japan; 2 Department of Pathology and Experimental Medicine, Kumamoto University, Kumamoto 860-8556, Japan; 3 Department of Gastroenterological Surgery, Graduate School of Medical Science, Kumamoto University, Kumamoto 860-8556, Japan

**Keywords:** LINE-1, SLIT2, MAL, IGFBP7, pyrosequencing, methylation

## Abstract

Genome-wide DNA hypomethylation and gene hypermethylation play important roles in instability and carcinogenesis. Methylation in long interspersed nucleotide element 1 (LINE-1) is a good indicator of the global DNA methylation level within a cell. Slit homolog 2 (SLIT2), myelin and lymphocyte protein gene (MAL) and insulin-like growth factor binding protein 7 (IGFBP7) are known to be hypermethylated in various malignancies. The aim of the present study was to assess the precise methylation levels of LINE-1, SLIT2, MAL and IGFBP7 in non-small cell lung cancer (NSCLC) using a pyrosequencing assay. Methylation of all regions was examined in 56 primary NSCLCs using a pyrosequencing assay. Changes in mRNA expression levels of SLIT2, MAL and IGFBP7 were measured before and after treatment with a demethylating agent. Methylation of these genes was also examined in 9 lung cancer cell lines using RT-PCR and a pyrosequencing assay. Frequencies of hypomethylation of LINE-1 and hypermethylation of SLIT2, MAL and IGFBP7, defined by predetermined cut off values, were 55, 64, 46 and 54% in NSCLCs, respectively and exhibited tumor-specific features. The hypermethylation of all genes was well correlated with changes in expression. The methylation level and frequency of MAL were significantly higher in smokers and in patients without EGFR mutations. Through accurate measurement of methylation levels using pyrosequencing, hypomethylation of LINE-1 and hypermethylation of SLIT2, MAL and IGFBP7 were frequently detected in NSCLCs and associated with various clinical features. Analysis of the methylation profiles of these genes may, therefore, provide novel opportunities for the therapy of NSCLCs.

## Introduction

Despite the progress of multimodal therapies for non-small cell lung cancer (NSCLC) including surgery, radiotherapy, and chemotherapy, the prognosis of patients remains poor. Therefore, accurate molecular analyses are essential for the establishment of an optimal treatment modality for each NSCLC patient.

CpG islands stretch over 500–2000 bp. These regulatory elements are located within promoter regions or the first exons of genes (1). In a normal genome, most cytosines of CpG sites in CpG islands are unmethylated, while methylated in X chromosome inactivation. Gene silencing associated with aberrant methylation of tumor-suppressor genes (TSGs) is a common mechanism of tumorigenesis in NSCLC (2). Global genomic DNA hypomethylation also plays an important role in genomic instability during tumorigenesis (3). Thus, the mechanisms of both DNA hypermethylation of TSGs and global genomic hypomethylation should be elucidated in order to understand and manage NSCLC.

The methods used to measure methylation in cancer cells or tissues varies among methylation-specific PCR (MSP) (4), real-time MSP (5), direct sequencing followed by subcloning, or combined bisulfite restriction analysis (COBRA) (6). Although all are technically sound, have widespread appeal and are frequently reported in the literature (7–10), each has limitations. Since the primers used for MSP and real-time MSP are designed to detect methylation at all of the CpG sites in the binding sequence, amplicons are generated only when the CpG island being examined is completely methylated. Subcloning and COBRA procedures are labor intensive and are thus not suitable for analysis of numerous samples; the non-quantitative nature of these methods is also a drawback.

In such a situation, the development of a simple, easy and accurate methylation assay seems necessary. Pyrosequencing is a nonelectrophoretic nucleotide extension sequencing technology with various applications including quantitative methylation analysis (11). Pyrosequencing has previously been shown to be more precise than COBRA and MethyLight (12). We previously demonstrated that one bisulfite-treated DNA specimen can provide precise measurement of a gene’s methylation value using pyrosequencing (13). Since a single run of PCR pyrosequencing can provide a reasonably precise measurement of gene methylation in a given specimen, and the variation in gene methylation values between different tissue sections from one tumor is relatively small, the gene methylation level of a representative tissue sample is most likely similar to the level of the entire tumor.

In the present study, the methylation levels of long interspersed nucleotide element 1 (LINE-1), Slit homolog 2 (SLIT2), T-cell differentiation/myelin and lymphocyte protein gene (MAL), and insulin-like growth factor binding protein 7 (IGFBP7, also named insulin-like growth factor binding protein-related protein) were assessed using sodium bisulfite conversion and a PCR pyrosequencing assay. Since these genes are known to be TSGs in various tumors, their role in tumorigenesis of NSCLC needs to be clarified. We correlated our results with clinicopathological features of the NSCLCs to learn more about how methylation profiles differ in NSCLC subtypes.

## Materials and methods

### Patients and cell lines

Specimens were obtained from 56 serial patients who underwent thoracic surgery at Kumamoto University Hospital from 2010 to 2012. None of these patients underwent preoperative chemotherapy, radiotherapy, or chemoradiotherapy. Informed consent for the research was obtained from each patient. The study design was approved by the ethics review board of our university.

Eight NSCLC cell lines (HCC15, H63, H157, H460, HUT15, HUT29, HUT70 and PC10) and one SCLC cell line (SBC5) were used in this study. We established several of the cell lines used (14), and others were purchased, or generously donated by Dr Adi F. Gazdar of the University of Texas Southwestern Medical Center.

### 5-Aza-2′-deoxycytidine (5-aza-CdR) treatment

The 9 tumor cell lines were incubated in culture medium with 1 μM of the demethylating agent 5-aza-CdR (Sigma-Aldrich, USA) for 6 days, with replacement of the medium on days 1, 3 and 5. Cells were harvested and RNA was extracted at day 6 as previously described (15).

### Reverse transcriptase-PCR (RT-PCR)

A reverse transcriptase-PCR (RT-PCR) assay was used to examine mRNA expression. Total RNA was extracted from samples with TRIzol (Life Technologies, USA) following the manufacturer’s instructions. The RT reaction was performed on 4 μg of total RNA using Deoxyribonuclease I and the SuperScript II First-Strand Synthesis System with the oligo(dT) Primer System (Life Technologies), and aliquots of the reaction mixture were subsequently used for PCR amplification. Real-time RT-PCR was performed using SYBR Premix Ex Taq (Perfect Real-Time) (Takara, Japan) and Thermal Cycler Dice^®^ Real-Time System TP850 software. The results were analyzed using the comparative Ct method (ΔΔCt) to compare the relative expression of each target gene before and after 5-aza-CdR treatment according to the user manual, and the ratio (5-aza-CdR/mRNA) was obtained. GAPDH was coamplified with target genes and served as an internal standard. Primer sequences were identical to those of the endogenous human target genes as confirmed by a BLAST search. For all RNA examined, the Gene Accession numbers are listed in parentheses. The real-time RT-PCR primer sequences used were (sequences following F, forward primer sequences; R, reverse primer sequences): SLIT2 (NM_004787.1) F, GGTGTCCTCTGTGATGAAGAG and R, GTGTTTAGGACACACACCTCG; MAL (NM_002371.2) F, GCAAGACGGCTTCACCTACAG and R, GCAGAGTG GCTATGTAGGAGAACA; IGFBP7 (NM_001553.1) F, CAC TGGTGCCCAGGTGTACT and R, TTGGATGCATGGCAC TCACA; GAPDH (NM_002046.3) F, TGAACGGGAAG CTCACTGG and R, TCCACCACCCTGTTGCTGTA. We confirmed that genomic DNA was not amplified with these primers, because all sequences cross an intron.

### DNA extraction and pyrosequencing assay

Genomic DNA was obtained from primary tumors and nonmalignant tissues by digestion with proteinase K, and phenol/chloroform (1:1) extraction using PureLink™ Genomic DNA kits (Invitrogen, USA).

DNA was treated with sodium bisulfite using EpiTect Bisulfite kits according to the manufacturer’s instructions (Qiagen, USA). Subsequent PCR and pyrosequencing for each gene was performed using the PyroMark kit (Qiagen) as described previously (16). The PCR conditions were 45 cycles of 95°C for 20 sec, 50°C for 20 sec and 72°C for 20 sec, followed by 72°C for 5 min. The biotinylated PCR products were purified and denatured prior to pyrosequencing with the Pyrosequencing Vacuum Prep Tool (Qiagen) in the PyroMark Q96 MD System (Qiagen). The nucleotide dispensation orders were: LINE-1 (X58075), ACTCAGTGTGTCAGTCAGTTAGTCTG; SLIT2, GTCGTCGTTGATTAGAGTGATCTGTCG; MAL, ATGTC GTCATGATAGTCGAGTTCGTCG; IGFBP7, GTCGTCGA TGTAGTTGTCG. The amount of C relative to the sum of the amounts of C and T at each CpG site was calculated as a percentage (i.e., 0–100). The average of the relative amounts of C in the CpG sites was used as the overall methylation level of each gene in a given tumor.

Detection of epidermal growth factor receptor (EGFR) gene mutations was performed using the Cycleave method as reported previously (17).

### Statistical analysis

The Fisher’s exact test and Mann-Whitney U test were applied to assess the association between categorical variables. Statistical significance was defined as a P-value <0.05. All P-values were two-sided. To determine the appropriate methylation cut-off value, each methylation level was subdivided into two cohorts using receiver operating characteristic (ROC) curve analysis (18). All statistical analyses were performed using SPSS 16.0 for Windows (SPSS, Inc., USA).

## Results

### Aberrant methylation of LINE-1, SLIT2, MAL and IGFBP7 in primary tumors

**Figure 1:**
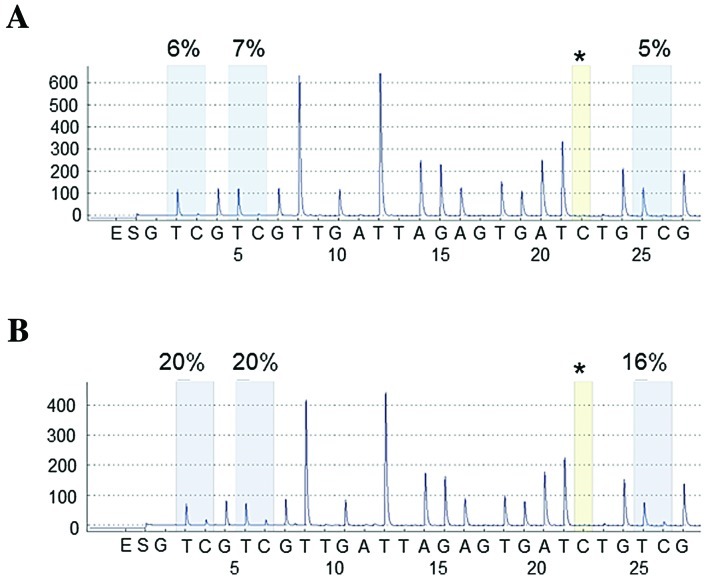
Measurement of the SLIT2 methylation level using pyrosequencing. (A) SLIT2 unmethylated non-malignant lung tissue (methylation level, 6%). (B) SLIT2 methylated tumor (methylation level, 18.7%). The percentages are the proportion of C at each CpG site after bisulfite conversion, and the methylation level of each CpG site is estimated by the proportion of C (%). An overall SLIT2 methylation level is calculated as the average of the proportion of C (%) at the 3 CpG sites. The asterisks indicate no residual C at the non-CpG site, ensuring complete bisulfite conversion.

We examined the methylation level of LINE-1, SLIT2, MAL and IGFBP7 in 56 NSCLC tissues and matched non-malignant lung tissues, with representative cases shown in Fig. 1. Tumor tissues showed significantly lower levels of LINE-1 methylation when compared with matched non-malignant lung tissues (P<0.0001). In contrast, methylation levels of SLIT2, MAL and IGFBP7 in tumor samples were higher when compared with levels in matched non-malignant lung tissues (P<0.0001, P=0.0010 and P<0.0001, respectively) (Table I). These data indicate that aberrant methylation was a tumor-specific event in NSCLCs.

**Figure 2:**
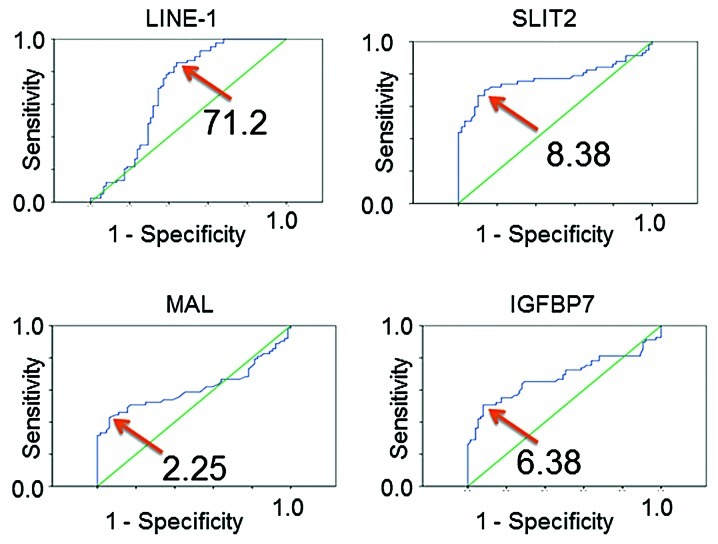
Receiver operating characteristic (ROC) curve analysis for predicting aberrant methylation. To determine the appropriate cut-off value, each methylation level was subdivided into two cohorts (tumor tissue and non-malignant lung tissue) using ROC curve analysis.

Studies that define cut-off values of aberrant methylation are important as they are useful for further discussion or cancer screening. Therefore, ROC curve analyses were conducted to obtain cut-off values for methylation within each genomic region. As shown in Fig. 2, cut-off values were determined, and the frequencies of aberrant methylation in both tumor and matched non-malignant lung tissue were obtained (Table I). Comparisons of tumor tissue with matched non-malignant lung tissue indicated that aberrant methylation of LINE-1, SLIT2, MAL and IGFBP7 were all tumor-specific events (all P<0.0001), despite the fact that tumor tissues consist of mixtures of tumor cells and non-malignant cells.

We next compared the clinicopathologic features with values of the methylation level and frequencies of aberrant methylation of LINE-1, SLIT2, MAL and IGFBP7 in NSCLCs (Table II). The level of LINE-1 methylation was significantly lower in squamous cell carcinoma when compared with the level in adenocarcinoma (P=0.0001). The methylation level of SLIT2 was significantly higher in cases of chronic obstructive pulmonary disease (COPD) when compared with the level in non-COPD cases (P=0.05). Frequencies of MAL hypermethylation were significantly higher in men than in women (P=0.035). In addition, the methylation level and methylation frequency of MAL were both significantly higher in smokers than in non-smokers (P=0.037; P=0.032) and in patients without EGFR mutations (P=0.001; P=0.002) compared to patients with EGFR mutations.

### Aberrant methylation and mRNA expression of SLIT2, MAL and IGFBP7 in the cell lines

We next examined the methylation level of LINE-1, SLIT2, MAL and IGFBP7 in cell lines and determined whether the observed levels were aberrant. According to the cut-off values obtained from primary tumor sample analyses (Table I), LINE-1 hypomethylation was present in 9 out of 9 cell lines. Hypermethylation of SLIT2, MAL and IGFBP7 was present in 7 out of 9, 9 out of 9, and 7 out of 9 cell lines, respectively (Table III). Next, expression of SLIT2, MAL and IGFBP7 was examined by real-time RT-PCR before and after demethylation with 5-aza-CdR treatment. By calculating the ratios of gene expression for the two treatments, we determined that SLIT2, MAL and IGFBP7 expression was higher after 5-aza-CdR treatment in 4 out of 9, 8 out of 9 and 7 out of 9 cell lines, respectively (Table III). The overall concordances between gene expression ratios and methylation levels of SLIT2, MAL and IGFBP7 were 67, 89 and 78% in lung cancer cell lines. The observed discordance could not be dissolved even when we changed the cut-off value of methylation (data not shown). Finally, we compared the methylation level of LINE-1 with methylation levels of other genes. There was no significant correlation between LINE-1 methylation and methylation of other genes (data not shown).

## Discussion

LINE-1 methylation is a good indicator of cellular 5-methylcytosine levels, and thus is correlated with the global DNA methylation level of cells or tissues. Previously, it was reported that hypomethylation of LINE-1 measured by real-time MSP was a prognostic marker in stage IA NSCLC (19). In our study, we first measured LINE-1 methylation in NSCLC using pyrosequencing, and found that LINE-1 was hypomethylated at higher frequencies in tumor tissues when compared with the frequencies in non-malignant lung tissue of NSCLCs. Although the number examined was relatively small and the prognosis was not examined, we detected significantly lower methylation levels in squamous cell carcinomas as compared to adenocarcinomas. The role of LINE-1 hypomethylation in carcinogenesis may be different between squamous cell carcinoma and adenocarcinoma.

The re-expression of SLIT2, MAL and IGFBP7 that was observed in lung cancer cell lines after treatment with a demethylating agent exhibited good concordance with the methylation status, suggesting that expression of those genes was downregulated mainly by hypermethylation. However, differences in the methylation level of each gene did not correlate linearly with the changes in expression measured before and after demethylation. Moreover, the discordance between methylation and expression remained even after modifying the cut-off value of methylation levels. The expression of each gene may be regulated partially by other mechanisms such as histone acetylation, loss of heterozygosity or miRNA, or may be due to toxicity of the demethylating agent.

SLIT2 suppresses tumor growth by coordinating regulation of the β-catenin and PI3K/AKT pathways. The hypermethylation of SLIT2 in NSCLC was examined by MSP (20). We also conducted similar experiments, but were not able to obtain clear results (data not shown), indicating that MSP can be influenced by conditions of the experiments, laboratory or reagents. In a previous study, SLIT2 methylation was examined by genome-wide DNA methylation analysis, using a microarray, in squamous cell carcinoma of the lung (21). Since SLIT2 is a well-known TSG and may play an important role in NSCLC tumorigenesis, we used pyrosequencing to obtain accurate and quantitative measurements of SLIT2 methylation in NSCLCs. Our results indicated that SLIT2 was hypermethylated in both tumor tissues and tumor cell lines. In addition, SLIT2 methylation levels were significantly higher in COPD cases than in non-COPD cases. We previously examined the molecular influence of COPD on the pathogenesis of NSCLC, and found that the genetic profile of COPD-related NSCLC was quite different from that of non-COPD-related NSCLC (22). Aberrant methylation of SLIT2 may also play a different role between COPD and non-COPD NSCLCs.

MAL is a T-cell differentiation protein. The MAL gene, which was initially isolated and cloned in 1987, maps to the long arm of chromosome 2, encodes a 16.7-kDa membrane protein, and contains a CpG island (23). MAL has been shown to possess tumor-suppressor capabilities by suppressing motility, invasion and tumorigenicity and by enhancing apoptosis in esophageal cancer (24). Previous studies have indicated that MAL is frequently methylated and plays a critical role in tumorigenesis of various types of tumors (25–27). However, to date there have been no studies examining MAL methylation in NSCLC. We examined methylation using pyrosequencing. Although the aberrant methylation level of the MAL gene was not high in comparison to the other genes examined here, methylation was significantly higher in tumor tissue than in non-malignant lung tissue. Moreover, the frequency of MAL methylation was significantly higher in males than in females, and both methylation level and methylation frequency were significantly higher in smokers and patients without EGFR mutations than in non-smokers and patients with EGFR mutations. Thus, the hypermethylation of MAL may play an important role in tumorigenesis of NSCLC, particularly in male smokers.

IGFBP7 is expressed in normal epithelial cells and acts as a tumor suppressor by inducing apoptosis in various types of tumors (28–30). The IGFBP7 gene is aberrantly methylated in various tumors (31,32), and methylation was previously detected in 46 out of 90 lung cancers examined by MSP (33). In our study, we evaluated the methylation level of the IGFBP7 gene in NSCLC using pyrosequencing, and detected hypermethylation in 30 out of 56 NSCLCs. The frequency of IGFBP7 hypermethylation tha was measured was quite similar to that reported by Chen *et al*, although the methodology and population were different (33).

In summary, through accurate measurement of methylation levels using a pyrosequencing assay, hypomethylation of LINE-1 and hypermethylation of SLIT2, MAL, and IGFBP7 were frequently detected in NSCLCs. Furthermore, these aberrant methylation profiles were accompanied by distinct clinical features. Further basic and clinical studies are required to investigate the role of aberrant methylation in NSCLC. Ultimately, further research in this area can contribute to the establishment of personalized treatment for NSCLC.

## Figures and Tables

**Table I:** Aberrant methylation levels of genes tested and cut-off values obtained from ROC curves.

Genes	Cut-off value	Tumor tissue				Matched non-malignant lung tissue				P-value^b^	P-value^c^
	
		Median	Mean ± SD	Range	Methylation (%)^a^	Median	Mean ± SD	Range	Methylation (%)^a^		
LINE-1	71.2	71.0	67.9±10.0	35.0–80.4	31 (55)	73.4	73.8±2.9	68.0–86.5	9 (16)	<0.0001	<0.0001
SLIT2	8.38	11.0	12.3±7.6	3.4–42.6	36 (64)	6.1	6.6±2.0	3.4–11.8	8 (14)	<0.0001	<0.0001
MAL	2.25	2.0	2.5±1.6	1.0–8.2	26 (46)	1.8	1.8±0.3	1.0–2.6	4 (7)	0.0010	<0.0001
IGFBP7	6.38	6.6	7.9±4.2	2.1–19.1	30 (54)	4.9	5.1±1.0	3.4–9.3	3 (5)	<0.0001	<0.0001

a Number and percentage of aberrant methylation determined by cut-off value.

b P-value obtained from paired t-test by comparing the methylation levels between tumors and matched non-malignant lung tissues.

c P-value obtained from Fisher’s exact probability test by comparing the frequencies of aberrant methylation.

**Table II:** Aberrant methylation levels and frequencies of LINE-1, SLIT2, MAL and IGFBP7 in NSCLC.

Patient characteristics	56 cases	LINE-1			P-value^a^ P-value^b^	SLIT2			P-value^a^ P-value^b^	MAL			P-value^a^ P-value^b^	IGFBP7			P-value^a^ P-value^b^
			
		Mean	n	%		Mean	n	%		Mean	n	%		Mean	n	%	
Age^c^ (years)																	
<72	27	70.4	15	56	NS	11.1	17	63	NS	2.51	13	48	NS	7.93	14	52	NS
≥72	29	65.6	16	55	NS	13.4	19	66	NS	2.57	13	45	NS	7.83	16	55	NS
Gender																	
Male	30	67.8	16	53	NS	11.8	20	67	NS	2.75	18	60	NS	8.75	19	63	NS
Female	26	68.1	14	54	NS	12.9	16	62	NS	2.31	8	31	0.035	6.88	11	42	NS
Smoking history																	
Smoker	32	67.1	18	56	NS	12.5	20	63	NS	2.93	19	59	0.037	8.72	20	63	NS
Never smoker	24	69.1	13	54	NS	12.0	16	67	NS	2.03	7	29	0.032	6.76	10	42	NS
COPD																	
(+)	21	66.3	11	52	NS	14.8	15	71	0.05	2.89	10	48	NS	9.03	14	67	NS
(−)	35	68.9	20	57	NS	10.7	21	60	NS	2.34	16	46	NS	7.19	16	46	NS
Histology																	
ADC	47	70.1	25	53	0.0001	12.2	32	68	NS	2.44	21	45	NS	8.09	26	55	NS
SCC	9	56.7	6	67	NS	12.5	4	44	NS	3.08	5	56	NS	6.79	4	44	NS
p-stage																	
IA	31	69.5	15	48	NS	11.1	18	58	NS	2.44	12	39	NS	7.90	17	55	NS
IB-III	25	66.0	16	64	NS	13.7	18	72	NS	3.32	14	56	NS	7.86	13	52	NS
EGFR mutation^d^																	
(+)	17	68.0	12	71	NS	11.5	13	76	NS	1.67	3	18	0.001	7.34	8	47	NS
(−)	18	69.7	9	50	NS	16.1	15	83	NS	3.61	13	72	0.002	9.41	13	72	NS

Mean, mean value of methylation level in tumor tissue; n, number of methylated cases according to the cut-off value of the ROS curve; NS, not significant; COPD, chronic obstructive pulmonary disease; ADC, adenocarcinoma; SCC, squamous cell carcinoma.

a P-value, methylation levels were compared by unpaired Student t-test.

b P-value, methylation frequencies were compared by Fisher’s exact probability test.

c Divided into 2 groups by median age.

d Thirty-five cases were examined.

**Table III:** Aberrant methylation and expression change ratio of lung cancer cell lines.

	LINE-1	SLIT2		MAL		IGFBP7	
				
Cell line	Methylation	Methylation	5-Aza-CdR/mRNA	Methylation	5-Aza-CdR/mRNA	Methylation	5-Aza-CdR/mRNA
SBC5	48.5↓	72.5↑	2.46	93.5↑	37.5	98.0↑	69.00
HCC15	33.4↓	44.6↑	1.73	6.4↑	23.5	7.6↑	7.84
H63	33.6↓	3.50	0.40	3.4↑	3.37	35.7↑	0.47
H157	40.4↓	22.8↑	1.91	26.4↑	30.0	64.7↑	1.26
H460	46.7↓	10.1↑	0.09	11.2↑	25.0	52.7↑	2.57
HUT15	29.1↓	28.4↑	0.33	4.5↑	2.40	4.7	0.54
HUT29	36.1↓	2.35	1.00	2.6↑	1.20	3.3	1.72
HUT70	69.6↓	83.0↑	4.14	3.8↑	25.9	95.6↑	5.82
PC10	14.4↓	75.7↑	0.63	6.6↑	1.00	6.8↑	2.23

Both up and down arrows indicate that the level exceeds the normal level as determined by ROC curve analysis.

## Abbreviations

LINE-1: long interspersed nucleotide element 1
SLIT2: slit homolog 2
MAL: myelin and lymphocyte protein gene
IGFBP7: insulin-like growth factor binding protein 7
NSCLC: non-small cell lung cancer
